# Enhancing SART Validity by Statistically Controlling Speed-Accuracy Trade-Offs

**DOI:** 10.3389/fpsyg.2013.00265

**Published:** 2013-05-13

**Authors:** Paul Seli, Tanya R. Jonker, James Allan Cheyne, Daniel Smilek

**Affiliations:** ^1^Department of Psychology, University of WaterlooWaterloo, ON, Canada

**Keywords:** SART, speed-accuracy trade-off, attention, sustained attention

## Abstract

Numerous studies focused on elucidating the correlates, causes, and consequences of inattention/attention-lapses employ the Sustained Attention to Response Task (SART), a GO-NOGO task with infrequent withholds. Although the SART has become popular among inattention researchers, recent work has demonstrated its susceptibility to speed-accuracy trade-offs (SATOs), rendering its assessment of inattention problematic. Here, we propose and illustrate methods to statistically control for the occurrence of SATOs during SART performance. The statistical solutions presented here can be used to correct standard SART-error scores, including those of already-published data, thereby allowing researchers to re-examine existing data, and to more sensitively evaluate the validity of earlier conclusions.

Attending to a given task is often important for successful task performance. For example, while driving, it is critical to pay attention to traffic signals, pedestrians, and other vehicles to minimize the risk of an accident. Although attention is undeniably important in various everyday tasks (such as driving), people nevertheless commonly experience lapses of attention while completing these tasks, leading them to, for example, arrive at a location with no memory of the drive to that location because of a failure of encoding during the attention lapse. As one might imagine, attention failures can lead to a host of dangerous scenarios, producing accidents, and errors in various contexts (e.g., Parker et al., 1995; Knowles and Tay, 2002). In addition to having serious implications in safety and critical situations, inattention can negatively impact learning in educational settings (see Smallwood et al., 2007). For example, lapsing attention has been shown to produce decrements in both reading comprehension (Schooler et al., 2004; Smallwood et al., 2008) and memory for lecture material (Risko et al., 2012). Thus, understanding the causes and consequences of attention failures is of great practical and theoretical importance to researchers because an improved understanding of these variables could potentially lead to methods of remediation and in turn, a reduction in the negative consequences of lapsing attention.

Indeed, over recent years, there have been increasing efforts to better understand attention failures in both real-world (e.g., Kane et al., 2007; Killingsworth and Gilbert, 2010; Risko et al., 2012) and laboratory settings (e.g., Manly et al., 1999; Strayer et al., 2003; Cheyne et al., 2009; Carriere et al., 2010; Mrazek et al., 2011). A considerable number of studies investigating inattention (e.g., Manly et al., 1999, 2004; Farrin et al., 2003; Smallwood et al., 2004a,b, 2009; O’Connell et al., 2008; Chan et al., 2009; Cheyne et al., 2009; Christoff et al., 2009; McVay and Kane, 2009; Carriere et al., 2010; Mrazek et al., 2012) have employed a common GO-NOGO task, known as the Sustained Attention to Response Task (SART; Robertson et al., 1997), to index attention failures. Whereas many sustained attention tasks require participants to withhold responses to frequently presented NOGO stimuli and to respond as quickly as possible to an infrequently presented GO stimulus, the SART reverses this common procedure by requiring participants to respond as quickly as possible to frequently presented GO stimuli and to withhold responses to an infrequently presented NOGO stimulus. Incorrect responses to the NOGO stimulus (i.e., errors of commission) are the primary measure of interest yielded by the SART, and have been used as an index of attention failures (with more errors of commission indicating more attention failures). In addition, other commonly-used measures of inattention yielded by the SART include errors of omission (failures to press to GO stimuli) and anticipations (very fast GO-trial responses) (Cheyne et al., 2009). A major appeal of the SART over traditional vigilance tasks is its sensitivity to moment-to-moment fluctuations in attention-to-task – given that it provides a behavioral measure on all GO trials – independent of long-term attention decrements (Robertson and O’Connell, 2010). Thus, given both the sensitivity and the brevity of the SART, it perhaps comes as no surprise that this task has been used in a considerable number of laboratory studies investigating inattention.

External validity of the SART has been provided by various studies showing that its errors of commission are associated with (1) self-reported attentional failures in everyday life (assessed via the Cognitive Failures Questionnaire; Robertson et al., 1997; Smilek et al., 2010), and (2) self-reported attention-related errors (assessed via the Attention-Related Cognitive Errors Scale; Cheyne et al., 2006; Smilek et al., 2010). Recently, however, concerns have been expressed regarding the possible influence of speed-accuracy trade-offs (SATOs) in the SART; more specifically, about whether the errors of commission observed in the SART are reflective of individual differences in response-speed strategies rather than attention failures, *per se* (e.g., Helton, 2009; Helton et al., 2009; Seli et al., 2012b,c). Providing strong support for the claim that the SART is influenced by SATOs, it was recently observed (Seli et al., 2012c) that SART errors are a systematic function of response speed, with slower responses producing fewer errors, suggesting that SART errors are not reflective solely of attention failures, but are also significantly influenced by individual differences in SATOs (see also Peebles and Bothell, 2004; Seli et al., 2012a,b).

Researchers (e.g., Robertson et al., 1997; Manly et al., 2002; Mullins et al., 2005) have tended to assume that SART errors constitute a single systematic effect (attention lapses) plus a non-systematic (random) effect. The present argument, however, is that SART errors are composed of two systematic effects (attention lapses and SATO effects) plus a random effect, and our purpose is to encourage the use of analytic methods using RT to separate attention lapses from SATO effects.

To avoid the problems surrounding the confounding influence of SATOs on SART errors, we recently suggested that researchers investigating inattention employ tasks that are not susceptible to SATOs (e.g., the Sustained Metronome-Modulated Attention to Response Task: SMMART: Seli et al., 2012c; or the Metronome Response task: MRT; Seli et al., 2013). Alternative solutions to the SATO problem, considered here, involve techniques to statistically control for the effect of response speed on errors of commission. A critical advantage of these statistical solutions is that they can be used to correct SART data that have already been published when measures of both errors and response times (RTs) are available, allowing researchers to deconfound and re-examine existing data, which will enable them to more carefully assess the validity of their earlier conclusions.

Here we present three statistical methods with which researchers can analyze (or reanalyze) SART-error data to provide a deconfounded measure of attention failures. The first method involves statistically controlling for the influence of SART RTs on SART errors (and vice versa) by simultaneously entering both of these measures as predictors of a dependent variable of interest in a regression analysis; this method can be used for correlational analyses involving SART data. The second and third methods, outlined below, can be used when researchers want to test the difference(s) in SART errors across conditions. Specifically, the second method involves conducting an analysis of covariance (ANCOVA) in which SART RTs are entered as a covariate, and the third method involves employing a structural equation model to examine the extent to which differences in SART-error rates across conditions are mediated by SART RTs.

To illustrate the first statistical method (i.e., the regression analysis in which RTs and errors are entered as simultaneous predictors of a dependent variable of interest), in Study 1, we conducted a regression analysis controlling for the influence of SART RTs on SART errors and examined the relation between the deconfounded SART-error measure and performance in a task, recently developed to measure attention lapses, called the Metronome Response task (MRT; Seli et al., 2013). In the MRT, participants are presented with a constant series of tones at a regular temporal interval and are instructed to respond synchronously (via button press) with the onset of each tone. Recent work using the MRT has shown that variance in responses to the metronome tones increases during periods of self-reported attentional disengagement relative to periods of on-task performance (Seli et al., 2013), thereby demonstrating the effectiveness of the MRT as a tool for indexing inattention. Thus, given that both the SART and MRT are believed to index inattention, from a theoretical standpoint, the measures yielded by these tasks ought to be related. However, under the assumption that SATOs produce confounded SART errors, this expected correlation might be attenuated. If this is the case, then we expect to observe a stronger correlation between MRT variance and SART errors after statistically controlling for the influence of SATOs on SART errors.

To examine this possibility, in Study 1 we had participants complete both the SART and the MRT and examined the relation between raw SART errors (i.e., errors for which response speed was not controlled) and MRT response variance. We then conducted a regression analysis to statistically control for the influence of SART RTs on SART errors and examined the relation between the deconfounded SART errors and the MRT response variance, seeking to determine whether this relation was stronger than the initially observed relation between raw SART errors and MRT variance.

To illustrate the second statistical method (i.e., conducting an ANCOVA in which SART RT is entered as a covariate), we reanalyzed previously reported SART data (Seli et al., 2012a,b) in which SATOs were apparent; these data sets allowed for comparisons of SART errors (both raw and deconfounded) across different conditions for both between-subjects (Reanalysis 1) and within-subjects (Reanalysis 2) designs.

Finally, in addition to illustrating how to control for the influence of SART RTs on SART errors using an ANCOVA, in Reanalysis 1, we illustrate the third statistical method in which a structural equation model is employed to test whether differences in SART-error rates across conditions are mediated by SART RTs.

## Study 1

### Method

#### Participants

We collected data from 118 undergraduates (36 males, 82 females, ages 18–25 years, *M* = 20.1) from the University of Waterloo, all of whom received course credit for their participation. All participants reported normal or corrected-to-normal vision. Of the 118 participants, 10 participants’ data were removed from all analyses because they failed to produce responses to at least 10% of either the SART or MRT trials (or both), indicating a failure to comply with instructions (see Seli et al., 2013). Hence, data from 108 participants were analyzed.

#### MRT

Each of the MRT trials started with 650 ms of silence, followed by the presentation of a tone (roughly 60dB) that lasted for 75 ms, and an additional 575 ms of silence (total trial duration = 1300 ms). Thus, the onset of the tone occurred at the midpoint of each 1300 ms trial. Participants first completed 18 practice trials to familiarize themselves with the task, after which they completed 900 experimental trials. Participants were instructed to respond synchronously with the onset of each tone by making a button press (spacebar) at the exact moment at which each tone was presented.

#### MRT measures

We calculated Rhythmic RT (RRTs; Seli et al., 2013) as the difference between the onset of each metronome tone and the associated button press. MRT RRT variances were then computed using a moving window of the preceding five trials across all trials throughout the task^1^ (excluding the first five trials of the task), and these variance scores were normalized using a natural logarithm transform (Seli et al., 2013). Finally, for each participant, we computed the average of the transformed variance scores across the moving window, producing what will be hereafter referred to as “overall MRT RRT variance.”

#### SART

On each SART trial, a single digit was presented for 250 ms in the center of the monitor, after which time an encircled “x” mask was presented for 900 ms (total trial duration = 1150 ms). For each block of nine trials, a single digit (1–9) was randomly chosen without replacement, and was presented in white on a black background. Thus, each of the digits appeared with equal frequency across the experimental trials. The digit sizes were randomly varied across all trials, with equal sampling of five possible font sizes (120, 100, 94, 72, and 48 points); this was to ensure that participants were not simply making their response decision on the basis of familiar features of a given stimulus. Participants were instructed to respond (by pressing the spacebar) to each GO digit (i.e., digits 1–2, and 4–9) and to withhold responses to each NOGO digit (i.e., 3). They were further instructed to place equal emphases on (1) responding as quickly as possible and (2) maintaining high accuracy. After 18 practice trials (containing 2 NOGO digits), participants completed 630 experimental trials (containing 70 NOGO digits; thus, 11% of all trials were NOGO trials).

#### SART measures

Mean RTs were calculated for GO-trial responses. If the participant did not make a response on a GO trial, this was coded as an omission. Responses made to the NOGO stimulus were coded as errors of commission.

#### Procedure

The order in which the tasks (i.e., SART and MRT) were completed was counterbalanced across conditions. Participants were randomly assigned to one of the two orders.

### Results and discussion

#### Relations between overall MRT RRT variance and SART errors

The Pearson Product-Moment correlations for mean SART errors and SART RT with overall MRT RRT variance were both non-significant, *r* = 0.13, *p* = 0.17, and *r* = 0.09, *p* = 0.19, respectively. SART errors and RT were, as is typical in SART research, highly correlated, *r* = 0.77, *p* < 0.01, though tolerance 0.41 and VIF (2.44) were in acceptable limits for the regression analysis reported. We next examined the relation between the SART errors residualized on SART RTs and MRT RRT variance. To do this, we carried out a multiple regression analysis predicting MRT RRT variance with SART errors and SART RTs. SART errors and RTs each made a unique contribution to the prediction of MRT RRT variance (semi-partial correlations were 0.31 and 0.29, respectively; both *p*s < 0.003), demonstrating that, when controlling for the influence of SART RTs on SART errors, the relation between these errors and MRT RRT variance was now significant, as theoretically expected. In addition, this result demonstrated that, when controlling for SATOs by removing the shared variance between SART RTs and errors, the RTs provide a relatively strong prediction of MRT RRT variance, indicating that SART errors and SART RTs each make an independent contribution to predicting inattention. This finding is consistent with the hypothesis that when SATO effects are controlled, SART errors are a consequence of attention lapses. The residual effects of SART RT, now also independent of SATO effects, likely reflect long attention-lapse RTs during periods without NOGO trials. This speculation is consistent with a previous finding that long RTs reliably precede SART omissions (i.e., failures to respond within the time constraints of the task; Cheyne et al., 2009), which have been found to be associated with ADHD (O’Connell et al., 2004; Johnson et al., 2007), and traumatic brain injury (Manly et al., 2003), as well as self-reported attention-lapses, and attention-related cognitive errors (Cheyne et al., 2009).

Given that both SART errors and MRT RRT variance are employed to index inattention, one expects them to be positively correlated. When, however, SART errors are confounded by SATOs, the correlation will be attenuated. Thus, although SART raw error scores were not significantly correlated with MRT RRT variance, by controlling for the influence of SART RTs on SART errors, in Study 1, we obtained a significant correlation between deconfounded SART errors and MRT RRT variance. Hence, failing to control for SATOs would lead one to commit a Type II error.

In Reanalyzes 1 and 2 (below), we attempted to broaden the application of statistically controlling for SATO in the SART by examining methods to control for SATOs in scenarios where researchers are interested in testing the difference(s) in SART-error rates across conditions (with, for example, *t*-tests and analyses of variance; ANOVAs). We examine this method of controlling for SATOs using both between-subjects (Reanalysis 1) and within-subjects (Reanalysis 2) data sets.

## Reanalyzes of Previously Published Data

### Reanalysis 1

In recent work (Seli et al., 2012b: Study 1), we explored the effects of SATOs in the SART by modifying such trade-offs through instructions. Specifically, we provided some participants with the standard SART instructions to “respond as quickly as possible (to GO digits) while maintaining a high level of accuracy (withholding to NOGO digits),” and other participants with instructions to “respond slowly and accurately.” Assuming that, (1) the standard SART-errors index inattention and (2) this measure is not susceptible to differences in response speed (i.e., SATOs), one would predict no differences in error rates across the two aforementioned conditions because the only difference between these conditions is the speed at which participants respond. However, if the SART is indeed susceptible to SATOs, then one would expect to observe differences in errors rates across these two conditions, with fewer errors produced by the group that was instructed to respond slowly and accurately. As expected, we observed significant increases in RTs for the “go-slow” condition relative to the standard instruction condition, and correspondingly, significantly fewer errors than in the standard condition, indicating a SATO.

Using these previously reported data (Seli et al., 2012b: Study 1), here we explored the hypothesis that the RT difference between the two conditions could completely explain the error difference. To do this, we conducted an ANCOVA to statistically control for the influence of RTs on errors in both the standard SART and go-slow SART conditions. In addition, we employed a structural equation model to test whether differences in SART-error rates across conditions are mediated by SART RTs.

### Method

#### Participants

All analyses for Reanalysis 1 use data from a previously reported study (Seli et al., 2012b: Study 1). Participants were 60 undergraduate students (12 males, 48 females, ages 18–23 years, *M* = 19.6) from the University of Waterloo, all of whom received course credit for their participation.

#### SART

The SART program and measures used in the Seli et al. (2012b) study were identical to those described in Study 1, with the exception of the instructions provided. Namely, half of the participants were provided the standard SART instructions to respond as quickly as possible while maintaining a high level of accuracy, whereas the other half were instructed to respond slowly and to maintain a high level of accuracy (see Seli et al., 2012b: Study 1). Participants were randomly assigned to one of these two conditions.

### Results and discussion

In the original (Seli et al., 2012b; Study 1) article, analyses of both RTs and errors across conditions included Block (first- and second-half) as a factor. However, given that this analysis goes beyond the goals of the present work, here we reanalyzed these data, this time excluding Block as a factor. The purpose of this analysis was to demonstrate that, when excluding Block as a factor, we still observe slower RTs and fewer errors produced by the group who was instructed to “go-slow” relative to those who were given the standard instructions, as was the case in the original report. After reanalyzing and demonstrating that this is indeed the case, we then applied our statistical solution to determine whether the difference in RTs accounted for the difference in error rates across the two conditions.

#### GO-trial RTs and NOGO errors for standard and go-slow conditions

To confirm our previous findings, this time without Block as a factor, we first examined the GO-trial RTs across the standard and go-slow conditions. An independent-samples *t*-test yielded a significant difference between these two conditions, *t*(58) = 3.23, SE = 26.75, *p* < 0.01, demonstrating that RTs were significantly faster in the standard (*M* = 351 ms) than the go-slow (*M* = 438 ms) SART condition.

Next, we examined the SART NOGO error rates across the standard and go-slow conditions. An independent-samples *t*-test yielded a significant difference in errors across these conditions, *t*(58) = 2.10, SE = 0.06, *p* < 0.05, with more errors produced in the standard (*M* = 0.45) than the go-slow (*M* = 0.32) condition.

#### Deconfounded NOGO errors for standard and go-slow conditions

Having demonstrated that the initial finding (Seli et al., 2012b) of slower RTs and fewer errors in the go-slow compared to the standard instruction condition remained when excluding Block as a factor, we next tested the hypothesis that the error difference observed between the two conditions was the result of different RTs across these conditions. To do this, we deconfounded the errors by statistically controlling for influence of GO-trial RTs on NOGO errors for each condition. Specifically, we conducted an ANCOVA with NOGO Errors as the dependent variable, Condition (standard versus go-slow instructions) as the fixed factor, and mean GO RTs as the covariate. The analysis failed to show a significant difference in errors across the two conditions, *F*(1,57) = 1.04, MSE = 0.02, *p* = 0.31, indicating that the initially observed difference in error rates across these two conditions was due to the different GO-trial RTs across the conditions.

**Figure 1:**
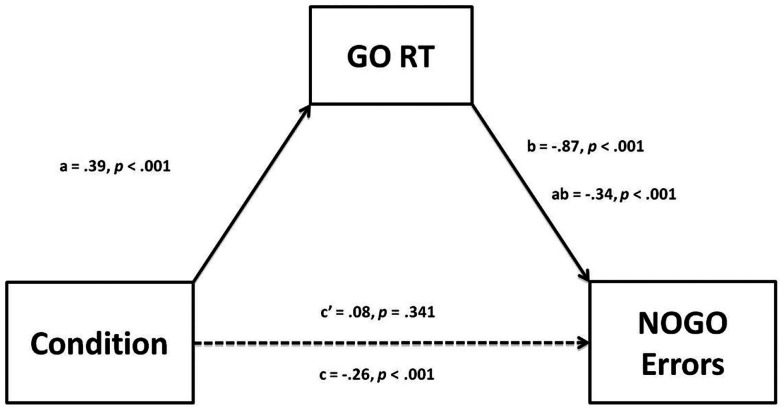
**Mediation model for Condition (Standard versus Go-Slow Instructions) with GO RT as a mediator of NOGO Errors**. a = direct effect of Condition on GO RT, b = direct effect of GO RT on NOGO errors, ab = indirect effect of Condition via GO RT on NOGO errors. c′ = direct effect of Condition on NOGO errors, c = total effect of Condition on NOGO errors.

In this case one can also employ structural equation modeling to test the hypothesis that the association between Condition (standard versus go-slow instructions) and NOGO Errors was mediated by GO RTs using both Condition and GO RT as predictors of errors. Mediation refers to a sequence of putative causal relations through which an independent variable exerts an effect on a dependent variable by influencing intervening (mediating) variables (e.g., Hayes, 2009). The mediation paradigm in the present reanalysis consists of three variables: an exogenous independent variable (Condition: standard vs. go-slow instructions, dummied as ±0.5), the mediator (GO RT) variable, and a dependent variable (NOGO errors). Among these three variables, there are three direct effects: a, b, and c′ and one indirect effect, ab (see Figure 1). To conclude mediation, when the independent variable and the mediator are entered into a regression analysis simultaneously, the indirect effect, ab, must be significantly different from zero. In the present case, however, we are interested not only in the indirect effect but also, and more particularly, in whether there remains a significant direct effect (c′) of Condition on NOGO errors when the mediated effect is removed from the total effect (c′ = c − ab, where c is the total effect of Condition on NOGO errors). An MLE regression analysis carried out using AMOS 21 (Arbuckle, 2012), employing bootstrapping (5000 samples) to establish confidence intervals, yielded both a significant indirect effect (ab), and a non-significant direct effect (c′) (see Figure 1). Thus, the effect of Condition on NOGO errors is fully accounted for by the indirect effect via GO RT. In some cases, it may be of interest to investigators to report the indirect effect size in terms of the unstandardized coefficient, which can be interpreted in the present case as the difference in proportion of errors between conditions attributable to the mediated effect. In the present study, the unstandardized coefficient is −0.16, indicating that participants made 16% fewer errors under the go-slow than under the standard condition attributable to the change in GO RT. Finally, it may have been noted by the astute reader that the indirect effect of Condition on NOGO errors in Figure 1 is larger than the total effect. This results from the positive (though non-significant) direct effect. Hence, the conflict between the positive direct effect and the negative indirect effect attenuates the total effect of Condition on NOGO errors. Had this direct positive effect been sufficiently strong, the total effect would have been zero and, had the analysis stopped at that point, one would have erroneously concluded that Condition had no effect on errors, when it would have had two significant, but opposing, conceptually meaningful effects. Such possibilities should alert the researcher to the importance of exploring the complex interdependencies among independent, dependent, and intervening variables.

In Reanalysis 2, we consider a case of controlling for SATO in the SART analysis for a within-subjects design.

### Reanalysis 2

In another recent study (Seli et al., 2012a; Study 1), which used a within-subjects design, we assessed sustained attention across modalities using (1) the standard visual SART and (2) a modified auditory SART, in which the digits and mask (white noise) were presented auditorily^2^. The results demonstrated a high level in error consistency across modalities, and, further, showed that GO RTs produced in the auditory SART were significantly slower than those produced in the visual SART, and that this slowing of RTs was associated with a decrease in NOGO errors compared to the visual SART.

Here, we reanalyzed these previously-reported data (Seli et al., 2012a) to again illustrate the effectiveness of controlling for the influence of SART RTs on SART errors. Specifically, we controlled for the influence of SART GO-trial RTs on errors in the visual and auditory versions of the SART, and then examined whether the errors still differed across these conditions.

### Method

#### Participants

All analyses for Reanalysis 2 use data from our previously reported study (Seli et al., 2012a: Study 1). Participants were 48 undergraduate students (17 males, 31 females, ages 18–24 years, *M* = 19.2) from the University of Waterloo. Participants were granted course credit for their participation.

#### Experimental conditions

All details of the visual SART (including the measures computed) were identical to those described in Study 1 of the present work, except that (1) the task consisted of 225 (as opposed to 630) trials and (2) the digits were presented for 300 ms (as opposed to 250 ms^3^). The auditory SART was identical to the visual SART except that digits and mask were presented auditorily.

#### Procedure

Each version of the SART consisted of 18 practice trials followed by 225 experimental trials.

### Results and discussion

As noted in footnote 2, the original study (Seli et al., 2012a) actually included three (not two) within-subjects conditions: (1) the standard visual SART, (2) an auditory version of the SART, and (3) an auditory-visual version of the SART. Thus, although in the original paper, we reported significantly slower RTs accompanied by significantly fewer errors in the auditory compared to the visual SART conditions, given that, here, we were not interested in the auditory-visual condition, we reanalyzed the RT and error data to demonstrate that the initially reported differences in RTs and errors across the visual and auditory conditions remained when we removed the auditory-visual condition data from our analyses. After having demonstrated that this was the case, we then moved on to employ the statistical solution (i.e., the ANCOVA) to determine whether the error difference across the two conditions could be accounted for by the RT difference across these conditions.

#### GO-trial RTs and errors for visual and auditory SART conditions

To confirm our previous findings, this time, in the absence of the auditory-visual data, we first examined the GO-trial RTs across only the auditory and visual SART conditions. A paired-samples *t*-test yielded a significant difference between these two conditions, *t*(47) = 5.84, SE = 11.17, *p* < 0.001, demonstrating that RTs were significantly faster in the visual (*M* = 367 ms) than the auditory (*M* = 432 ms) SART condition. We then examined the SART-NOGO-error rates across the auditory and visual SART conditions. A paired-samples *t*-test yielded a significant difference in errors across these conditions, *t*(47) = 3.53, SE = 0.02, *p* < 0.01, with more errors produced in the visual (*M* = 0.40) than the auditory (*M* = 0.34) SART condition. Thus, these results are suggestive of a SATO.

#### Deconfounded NOGO errors for visual and auditory SART conditions

Having demonstrated that the initially reported differences in RTs and errors across the auditory and visual SART conditions remained when excluding the auditory-visual SART condition data, we next assessed the hypothesis that the error differences observed between the auditory and visual SART conditions were the result of different GO-trial RTs across these conditions (i.e., a SATO). To do this, we deconfounded the errors for both conditions using an ANCOVA to statistically control for the influence of RTs on errors. Rather than using auditory and visual SART RTs as separate covariates, we computed the difference between these RTs and entered this value as a covariate. According to the SATO hypothesis, the difference in error rates between the two conditions is a consequence of the difference in RTs; thus, controlling for the difference in RTs across the auditory and visual SART conditions should eliminate the observed difference in error rates. To examine this hypothesis, we conducted a repeated-measures ANCOVA with Errors (for both the auditory and visual SART conditions) as the dependent variable, Condition (auditory vs. visual) as the within-subjects factor, and the difference in RTs across the conditions as the covariate. The analysis failed to produce a significant effect, *F*(1,46) = 1.63, MSE = 0.02, *p* = 0.21, indicating, as was the case in Reanalysis 1 of the present article, that the initially observed difference in error rates across the two SART conditions was a consequence of the different GO-trial RTs across these conditions.

## General Discussion

In the present work, we explored the utility of three methods to statistically control for the problematic effects of SATOs in the SART; a commonly used task in studies of inattention. In Study 1, we examined the correlation between SART errors and performance on a recently-developed task that indexes inattention (the MRT; Seli et al., 2013). Initially, before controlling for the effect of SATOs on SART errors, we failed to observe a significant correlation between the performance measures obtained from these two tasks. At first, this finding was somewhat surprising given that, theoretically, performance in these two tasks should be positively related since both tasks are believed to be sensitive to attentional disengagement. By controlling for the effects of SATOs on SART errors, however, we observed a significant partial correlation between SART errors and MRT performance, thereby supporting the claim that SATOs produce a confounded measure of errors in the SART (Peebles and Bothell, 2004; Helton, 2009; Helton et al., 2009; Seli et al., 2012a,b,c), and further, demonstrating the importance of controlling for SATOs in the SART.

Although, in Study 1, the regression analysis that controlled for SATOs in the SART proved to be effective, this particular method is useful only in situations where researchers are interested in examining SART data with correlational analyses. Thus, in Reanalyzes 1 and 2, we considered methods appropriate for between- and within-subjects data sets in which differences in error rates across two or more conditions are tested (using either *t-*tests or ANOVAs).

In Reanalysis 1, we reanalyzed previously reported between-subjects data from a recent study (Seli et al., 2012b; Study 1) in which the effects of SATO in the SART were explored by modifying SATO with an instructional manipulation. As expected, participants who were instructed to respond slowly and accurately produced significantly longer GO-trial RTs than those who were provided the standard SART instructions to respond as quickly and accurately as possible. Initially, without controlling for the effects of SATOs on SART errors, a significant difference in error rates was observed, with more errors in the standard group, which produced faster RTs than the go-slow group. However, after conducting an ANCOVA to statistically control for the differences in RTs across the two groups, the difference in error rates disappeared. We then employed a structural equation model to test whether the relation between condition and SART errors was mediated by RTs and found that this was indeed the case, thereby supporting the hypothesis that differences in RTs in the SART can produce dramatic differences in error rates, independent of actual attentional abilities. This finding further supported the advisability of controlling for SATO in the SART.

Finally, in Reanalysis 2, we reanalyzed previously reported data from a within-subjects design (Seli et al., 2012a) in which participants completed both a visual and auditory version of the SART. Once again, when controlling for the difference in RTs across the two conditions, the initially observed difference in error rates was eliminated.

Although SATOs have been well-documented in various sub-fields of Cognition (Woodworth, 1899; Garrett, 1922; Hick, 1952; see Pachella, 1974 and Sperling and Dosher, 1986, for reviews), these effects have largely been ignored in the attention-lapse and mind-wandering literatures. Methods of controlling for the influence of SATOs on SART errors is particularly important for the attention-lapse literature in cases where researchers attempt to modify sustained attention performance (e.g., attention training studies) because, otherwise, it cannot be determined whether the researchers are manipulating SATOs or attention. Thus, the proposed analyses are critical in such situations as they allow for the dissociation of response speed and SART errors, thereby allowing researchers to determine whether they have effectively altered attentional ability or response speed. Statistically controlling for SATOs is also important in investigations of different disorders (e.g., Schizophrenia, Chan et al., 2009; Affective disorders, Smallwood et al., 2007) because there is the possibility that individuals suffering from some disorders differ in their RTs in the SART, and as a consequence, error rates, independently of attentional ability. Although the SART has been used in a considerable number of studies investigating the attentional abilities of various disordered participants, results of the present studies suggest the possibility that the inferences drawn from these studies might need to be re-evaluated and adjusted. Of course, our participants were drawn from a particular population comprising undergraduate students, so we cannot readily conclude that our findings will generalize to other populations. Future research will be needed to evaluate this possibility.

It is worth noting that we do not intend to make the claim that results from all SART studies are invalid because of SATOs. Although here we demonstrate one such instance where failing to control for the influence of SATOs in the SART would produce incorrect conclusions (Study 1), this might not always be the case. Nevertheless, the results presented in this article suggest that, at least in some cases, controlling for SATOs will produce dramatically different results; thus, it is clearly important that researchers control for SATOs to enhance the validity of SART errors as a measure of sustained attention. As noted, one benefit of the statistical methods presented here is that they allow researchers to re-examine existing SART data in cases where RTs and error rates were obtained. Thus, we strongly encourage researchers who have used the SART to reanalyze their data while controlling for SATO effects and to report the results of their reanalyzed studies to enhance the clarity and validity of the inattention literature.

## Author Note

This research was supported by a Natural Sciences and Engineering Research Council of Canada (NSERC) discovery grant to Daniel Smilek and NSERC Vanier Canada Graduate Scholarships to Paul Seli and Tanya R. Jonker.

## Conflict of Interest Statement

The authors declare that the research was conducted in the absence of any commercial or financial relationships that could be construed as a potential conflict of interest.

## Footnotes

^1^The moving-window analysis was adopted because it retained the temporal component of the variance across the task. Simply computing the overall variance of the RRTs would not retain this temporal component.

^2^In Study 1 of the Seli et al. (2012a) paper, participants also completed an auditory-visual version of the SART that combined the features of both the auditory and visual SARTs. However, given that the errors rates and RTs in this condition were not significantly different from the auditory or visual conditions, we opted to exclude these data from the present analyses. Because we excluded the data from the auditory-visual condition, here, we reanalyzed the error and RT data across only the auditory and visual conditions.

^3^This was due to the fact that, at the standard 250 ms presentation rate, the auditory stimuli were difficult to comprehend, whereas at 300 ms, they were much easier to comprehend.

## References

[B1] Arbuckle J. L. (2012). Amos 21 Users Guide. Amos Development Corporation

[B2] Carriere J. S. A. Cheyne J. A. Solman G. J. F. Smilek D. (2010). Age trends for failures of sustained attention. Psychol. Aging 25, 569 10.1037/a0019363 20677878

[B3] Chan R. C. K. Wang Y. Cheung E. F. C. Cui J. Deng Y. Yuan Y. (2009). Sustained attention deficit along the psychosis proneness continuum: a study on the sustained attention to response task (SART). Cogn. Behav. Neurol. 22, 180–185 10.1097/WNN.0b013e3181b7ef84 19741328

[B4] Cheyne J. A. Carriere J. S. A. Smilek D. (2006). Absent-mindedness: lapses of conscious awareness and everyday cognitive failures. Conscious. Cogn. 15, 578–592 10.1016/j.concog.2005.11.009 16427318

[B5] Cheyne J. A. Solman G. J. F. Carriere J. S. A. Smilek D. (2009). Anatomy of an error: a bidirectional state model of task engagement/disengagement and attention-related errors. Cognition 111, 98–113 10.1016/j.cognition.2008.12.009 19215913

[B6] Christoff K. Gordon A. M. Smallwood J. Smith R. Schooler J. W. (2009). Experience sampling during fMRI reveals default network and executive system contributions to mind wandering. Proc. Natl. Acad. Sci. U.S.A. 106, 8719–8724 10.1073/pnas.0900234106 19433790 PMC2689035

[B7] Farrin L. Hull L. Unwin C. Wykes T. David A. (2003). Effects of depressed mood on objective and subjective measures of attention. J. Neuropsychiatry Clin. Neurosci. 15, 98–104 10.1176/appi.neuropsych.15.1.98 12556579

[B8] Garrett H. E. (1922). A Study of the Relation of Accuracy to Speed. Archives of Psychology

[B9] Hayes A. F. (2009). Beyond Baron & Kenny: statistical mediation analysis for the new millennium. Commun. Monogr. 76, 408–420 10.1080/03637750903310360

[B10] Helton W. S. (2009). Impulsive responding and the sustained attention to response task. J. Exp. Clin. Neuropsychol. 31, 39–47 10.1080/13803390801978856 18608658

[B11] Helton W. S. Kern R. P. Walker D. R. (2009). Conscious thought and the sustained attention to response task. Conscious. Cogn. 18, 600–607 10.1016/j.concog.2009.06.002 19589699

[B12] Hick W. E. (1952). On the rate of gain of information. Q. J. Exp. Psychol. 4, 11–26 10.1080/17470215208416600

[B13] Johnson K. A. Robertson I. H. Kelly S. P. Silk T. J. Barry E. Dáibhis A. (2007). Dissociation of performance of children with ADHD and high-functioning autism on a task of sustained attention. Neuropsychologia 45, 2234–2245 10.1016/j.neuropsychologia.2007.02.019 17433378 PMC2000292

[B14] Kane M. J. Brown L. H. McVay J. C. Silvia P. J. Myin-Germeys I. Kwapil T. R. (2007). For whom the mind wanders, and when: an experience-sampling study of working memory and executive control in daily life. Psychol. Sci. 18, 614–621 10.1111/j.1467-9280.2007.01948.x 17614870

[B15] Killingsworth M. A. Gilbert D. T. (2010). A wandering mind is an unhappy mind. Science 330, 932–932 10.1126/science.1192439 21071660

[B16] Knowles D. Tay R. (2002). “Driver inattention: more risky than the fatal four?,” in Proceedings of the 2002 Road Safety Research, Policing and Education Conference (Adelaide, SA), 377–392

[B17] Manly T. Hawkins K. Evans J. Woldt K. Robertson I. H. (2002). Rehabilitation of executive function: facilitation of effective goal management of complex tasks using periodic alerts. Neuropsychologia 40, 271–281 10.1016/S0028-3932(01)00086-0 11684160

[B18] Manly T. Heutink J. Davidson B. Gaynord B. Greenfield E. Parr A. (2004). An electric knot in the handkerchief: “Content free cueing” and the maintenance of attentive control. Neuropsychol. Rehabil. 14, 89–116 10.1080/09602010343000110

[B19] Manly T. Owen A. M. McAvinue L. Datta A. Lewis G. H. Scott S. K. (2003). Enhancing the sensitivity of a sustained attention task to frontal damage: convergent clinical and functional imaging evidence. Neurocase 9, 340–349 10.1076/neur.9.4.340.15553 12925947

[B20] Manly T. Robertson I. H. Galloway M. Hawkins K. (1999). The absent mind: further investigations of sustained attention to response. Neuropsychologia 37, 661–670 10.1016/S0028-3932(98)00127-4 10390027

[B21] McVay J. C. Kane M. J. (2009). Conducting the train of thought: working memory capacity, goal neglect, and mind wandering in an executive-control task. J. Exp. Psychol. 35, 196–204 10.1037/a0014104 PMC2750806 19210090

[B22] Mrazek M. D. Chin J. M. Schmader T. Hartson K. A. Smallwood J. Schooler J. W. (2011). Threatened to distraction: mind-wandering as a consequence of stereotype threat. J. Exp. Soc. Psychol. 47, 1243–1248 10.1016/j.jesp.2011.05.011

[B23] Mrazek M. D. Smallwood J. Schooler J. W. (2012). Mindfulness and mind-wandering: finding convergence through opposing constructs. Emotion 12, 442–448 10.1037/a0026678 22309719

[B24] Mullins C. Bellgrove M. A. Gill M. Robertson I. H. (2005). Variability in time reproduction: difference in ADHD combined and inattentive subtypes. J. Am. Acad. Child Adolesc. Psychiatry 44, 169–176 10.1097/00004583-200502000-00009 15689730

[B25] O’Connell R. G. Bellgrove M. A. Dockree P. M. Lau A. Fitzgerald M. Robertson I. H. (2008). Self-alert training: volitional modulation of autonomic arousal improves sustained attention. Neuropsychologia 46, 1379–1390 10.1016/j.neuropsychologia.2007.12.018 18249419

[B26] O’Connell R. G. Bellgrove M. A. Dockree P. M. Robertson I. H. (2004). Reduced electrodermal response to errors predicts poor sustained attention performance in attention deficit hyperactivity disorder. Neuroreport 15, 2535–2538 10.1097/00001756-200411150-00021 15538190

[B27] Pachella R. G. (1974). “The interpretation of reaction time in information processing research,” in Human Information Processing: Tutorials in Performance and Cognition, ed. Kantowitz B. (New York: Halstead Press), 41–82

[B28] Parker D. Reason J. T. Manstead A. S. Stradling S. G. (1995). Driving errors, driving violations and accident involvement. Ergonomics 38, 1036–1048 10.1080/00140139508925170 29105607

[B29] Peebles D. Bothell D. (2004). “Modelling performance in the sustained attention to response task,” in Proceedings of the Sixth International Conference on Cognitive Modeling, 231–236

[B30] Risko E. F. Anderson N. Sarwal A. Engelhardt M. Kingstone A. (2012). Everyday attention: variation in mind wandering and memory in a lecture. Appl. Cogn. Psychol. 26, 234–242 10.1002/acp.1814

[B31] Robertson I. H. Manly T. Andrade J. Baddeley B. T. Yiend J. (1997). “Oops!”: performance correlates of everyday attentional failures in traumatic brain injured and normal subjects. Neuropsychologia 35, 747–758 10.1016/S0028-3932(97)00015-8 9204482

[B32] Robertson I. H. O’Connell R. (2010). “Vigilant attention,” in Attention and Time, eds Nobre K. Coull J. T. (Oxford: Oxford University Press), 79–88

[B33] Schooler J. W. Reichle E. D. Halpern D. V. (2004). “Zoning out during reading: evidence for dissociations between experience and metaconsciousness,” in Thinking and Seeing: Visual Metacognition in Adults and Children, ed. Levin D. T. (Cambridge, MA: MIT Press), 204–226

[B34] Seli P. Cheyne J. A. Barton K. R. Smilek D. (2012a). Consistency of sustained attention across modalities: comparing visual and auditory versions of the SART. Can. J. Exp. Psychol. 66, 44–50 10.1037/a0025111 21910522

[B35] Seli P. Cheyne J. A. Smilek D. (2012b). Attention failures versus misplaced diligence: separating attention lapses from speed-accuracy trade-offs. Conscious. Cogn. 21, 277–291 10.1016/j.concog.2011.09.017 22001770

[B36] Seli P. Jonker T. R. Solman G. J. F. Cheyne J. A. Smilek D. (2012c). A methodological note on evaluating performance in a sustained-attention-to-response task. Behav. Res. Methods10.3758/s13428-012-0266-1 23055171

[B37] Seli P. Cheyne J. A. Smilek D. (2013). Wandering minds and wavering rhythms: linking mind wandering and behavioral variability. J. Exp. Psychol. Hum. Percept. Perform. 39, 1–5 10.1037/a0030954 23244046

[B38] Smallwood J. Davies J. B. Heim D. Finnigan F. Sudberry M. O’Connor R. (2004a). Subjective experience and attentional lapse: task engagement and disengagement during sustained attention. Conscious. Cogn. 13, 657–690 10.1016/j.concog.2004.07.004 15522626

[B39] Smallwood J. M. O’Connor R. C. Sudbery M. V. Haskell C. Ballantine C. (2004b). The consequences of encoding information on the maintenance of internally generated images and thoughts: the role of meaning complexes. Conscious. Cogn. 13, 789–820 10.1016/j.concog.2004.07.004 15522632

[B40] Smallwood J. Fishman D. J. Schooler J. W. (2007). Counting the cost of an absent mind: mind wandering as an underrecognized influence on educational performance. Psychon. Bull. Rev. 14, 230–236 10.3758/BF03194057 17694906

[B41] Smallwood J. McSpadden M. Schooler J. W. (2008). When attention matters: the curious incident of the wandering mind. Mem. Cognit. 36, 1144–1150 10.3758/MC.36.6.1144 18927032

[B42] Smallwood J. M. Fitzgerald A. Miles L. K. Phillips L. H. (2009). Shifting moods, wandering minds: negative moods lead the mind to wander. Emotion 9, 271–276 10.1037/a0014855 19348539

[B43] Smilek D. Carriere J. S. A. Cheyne J. A. (2010). Failures of sustained attention in life, lab, and brain: ecological validity of the SART. Neuropsychologia 48, 2564–2570 10.1016/j.neuropsychologia.2010.05.002 20452366

[B44] Sperling G. Dosher B. A. (1986). “Strategy and optimization in human information processing,” in Handbook of Perception and Performance, Vol. 1, eds Boff K. Kaufman L. Thomas J. (New York: Wiley), 2.1–2.65

[B45] Strayer D. L. Drews F. A. Johnston W. A. (2003). Cell phone-induced failures of visual attention during simulated driving. J. Exp. Psychol. Appl. 9, 23–32 10.1037/1076-898X.9.1.23 12710835

[B46] Woodworth R. S. (1899). The accuracy of voluntary movement. Psychol. Monogr. 3, 2 10.1037/h0092992

